# Modeling virus exposure during male to female transmission of HIV-1

**DOI:** 10.1186/1742-4690-9-S2-O59

**Published:** 2012-09-13

**Authors:** A Carias, M McRaven, M Anderson, RS Veazey, TJ Hope

**Affiliations:** 1 Tulane National Primate Research Center, Covington, LA, USA; 2 Northwestern University, Feinberg School of Medicine, Chicago, IL, USA

## Background

Previously, we illustrated that HIV is able to penetrate the intact female genital mucosal barrier. We found that HIV penetrates to depths where target cells reside. Using data obtained from tissues of more than 40 donors, we are able to generate an average for the ability of HIV to penetrate the mucosal. Using these averages we can extrapolate the number of HIV particles penetrating the mucosa per coital act for male to female HIV transmission for both acute and chronic infections.

## Methods

Human cervical explants and macaque genital tracts were exposed to photoactivatable (PA-GFP) HIV. Rhesus macaques were inoculated intravaginally and genital tissues were removed 4 hours post-inoculation and dissected into relevant tissue specimens. Samples were snap frozen, sectioned, stained accordingly, and imaged for virus penetration.

## Results

We observed HIV penetration of the stratified squamous epithelium per image field to be 1.21 penetrating virions/scan when applied at 500ng/ml p24. Virus entered the tissue by diffusion/adsorption as revealed by fluid phase markers. Using these data, coupled with published determinations of average vaginal surface area and semen viral loads, we estimate that up to 14,465 virions penetrate the mucosal barrier during transmission during acute phase infection and approximately 18 virions during HIV chronic infection. We estimate that approximately 1-2 cm2 of the endocervix is exposed without a thick mucus barrier allowing 1-2 virions to penetrate.

## Conclusion

This model is consistent with known characteristics of transmission. Having a very low number of virions with the ability to initiate transmission is consistent with the low transmission rate seen under conditions of chronic infection in monogamous heterosexual couples. Data is also consistent with the knowledge that typically, only 1 virion establishes systemic infection after male to female HIV transmission. These results provide a biological description of the functional virus dose of HIV during transmission.

